# Prevalence of asymptomatic valvular heart disease in the elderly population: a community-based echocardiographic study

**DOI:** 10.1093/ehjci/jeae127

**Published:** 2024-06-26

**Authors:** Vasiliki Tsampasian, Cristian Militaru, Sathish Kumar Parasuraman, Brodie L Loudon, Crystal Lowery, Amelia Rudd, Janaki Srinivasan, Satnam Singh, Girish Dwivedi, Gnanadevan Mahadavan, Dana Dawson, Allan Clark, Vassilios S Vassiliou, Michael P Frenneaux

**Affiliations:** Norwich Medical School, University of East Anglia, Rosalind Franklin Road, Norwich NR4 7UQ, UK; Department of Cardiology, Norfolk and Norwich University Hospital, Colney Lane, Norwich NR4 7TJ, UK; Norwich Medical School, University of East Anglia, Rosalind Franklin Road, Norwich NR4 7UQ, UK; Norwich Medical School, University of East Anglia, Rosalind Franklin Road, Norwich NR4 7UQ, UK; Norwich Medical School, University of East Anglia, Rosalind Franklin Road, Norwich NR4 7UQ, UK; Norwich Medical School, University of East Anglia, Rosalind Franklin Road, Norwich NR4 7UQ, UK; University of Aberdeen, Aberdeen, UK; Aberdeen Royal Infirmary, Foresterhill Health Campus, Foresterhill Road, Aberdeen AB25 2ZN, UK; Royal Bournemouth Hospital, Castle Lane East, Bournemouth BH7 7DW, UK; Harry Perkins Institute of Medical Research, The University of Western Australia, Fiona Stanley Hospital, 5 Robin Waren Dr, Murdoch, WA 6150, Australia; Central and Northern Adelaide Health Services, Eastwood, SA 5063, Australia; University of Aberdeen, Aberdeen, UK; Norwich Medical School, University of East Anglia, Rosalind Franklin Road, Norwich NR4 7UQ, UK; Norwich Medical School, University of East Anglia, Rosalind Franklin Road, Norwich NR4 7UQ, UK; Department of Cardiology, Norfolk and Norwich University Hospital, Colney Lane, Norwich NR4 7TJ, UK; Department of Cardiology, Royal Brompton Hospital, Imperial College London, Sydney Street, London SW3 6NP, UK

**Keywords:** valvular heart disease, asymptomatic, silent, echocardiography, epidemiology

## Abstract

**Aims:**

With an ageing population, the presence of asymptomatic valvular heart disease (VHD) in the community remains unknown. The aim of this study is to determine the prevalence and associated factors of asymptomatic VHD in individuals ≥60 years old and to evaluate the feasibility of echocardiographic screening for VHD in this population.

**Methods and results:**

This was a prospective cohort study conducted between 2007 and 2016 in the UK. Asymptomatic patients with no prior indication for echocardiography were invited to participate and evaluated with a health questionnaire, clinical examination, and transthoracic echocardiography. A total of 10,000 individuals were invited through their general practices. A total of 5429 volunteered to participate, of whom 4237 were eligible for inclusion. VHD was diagnosed in more than a quarter of patients (28.2%). The most common types of VHD were regurgitation of the tricuspid (13.8%), mitral (12.8%), and aortic (8.3%) valves (trivial regurgitation was not included). The rate of prevalence of clinically significant VHD was 2.4% (2.2% moderate and 0.2% severe), with mitral and aortic regurgitation being the most common. The only parameter associated with significant VHD was age (odds ratio 1.07 per 1 year increment, 95% confidence interval 1.05–1.09, *P* < 0.001). The number needed to scan to diagnose one clinically significant case of VHD is 42 for individuals ≥60 and 15 for those ≥75 years old.

**Conclusion:**

Asymptomatic VHD is present in a significant proportion of otherwise healthy individuals without known VHD over 60 years old. Age is strongly associated with an increased incidence of significant VHD.

## Graphical Abstract

**Graphical Abstract:**
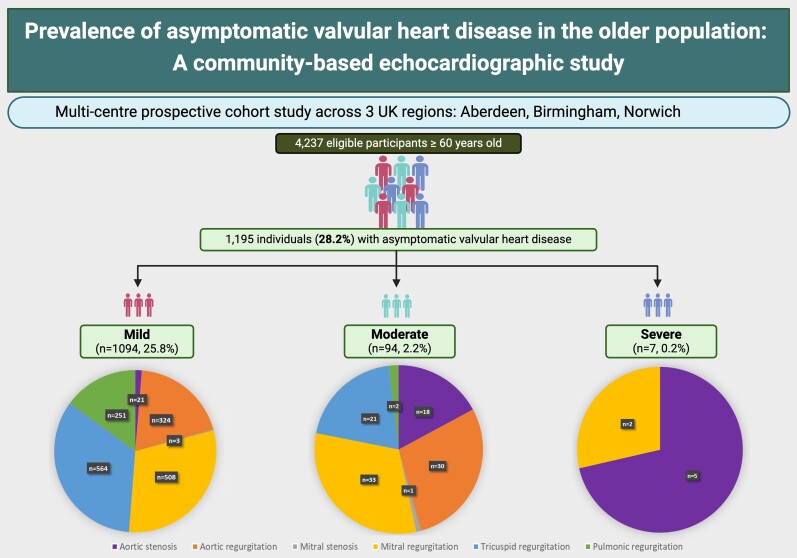

**See the editorial comment for this article ‘Valvular heart disease: the oncoming tsunami’, by K.L. Chan, https://doi.org/10.1093/ehjci/jeae146.**

## Introduction

Valvular heart disease (VHD) is an important cause of morbidity and mortality worldwide, posing a significant economic burden on healthcare services.^1,2^ By 2050, it is expected that the population above 60 years old will double, and above 80 years old will triple.^3^ With increasing age, the valves are subject to anatomical and functional deterioration,^4–6^ making valve disease more common in the elderly.^7,8^ Furthermore, with increasing life expectancy, VHD is likely to become even more prevalent in the future.

The diagnosis and evaluation of VHD mostly relies on transthoracic echocardiography, but referral for echocardiography is usually based on symptoms or abnormal cardiac auscultation. This can be challenging in the elderly, because mild symptoms may be masked by reduced physical activity and impaired mobility. In addition, cardiac auscultation has demonstrated limited accuracy for the detection of VHD in asymptomatic elderly patients and is thus a poor diagnostic screening tool in primary care.^9^ As a result, there may be a significant proportion of VHD that remains undiagnosed in this population. The feasibility of echocardiography as a large-scale screening tool for VHD in asymptomatic elderly people has not been investigated.

The diagnosis of VHD before the onset of symptoms can have several advantages. First, it would enable the initiation of appropriate medical therapy and risk factor modification and also enrol patients in a close follow-up programme. Secondly, in the case of asymptomatic severe VHD, it has been shown that an early surgery strategy in some valvular lesions can be associated with a significant long-term reduction in cardiac mortality and morbidity when compared with conventional strategies.^10–15^ In support of these findings, both the American and the European guidelines for the management of VHD also recommend intervention, based on echocardiographic criteria alone, for certain patients with asymptomatic VHD.^16^ We therefore investigated the prevalence of asymptomatic VHD in healthy subjects over the age of 60, in a large community study, and the potential role of routine echocardiographic screening in this population.

## Methods

This study was conducted between 2007 and 2016 in the UK.^17^ A total of 10,000 people over 60 years of age were invited to participate through general practices in three areas of the UK (West Midlands, Aberdeenshire, and Norfolk) by selecting practices representing the demographics of the UK population. The objective of this study was to establish the prevalence of *asymptomatic* VHD and determine the feasibility of echocardiography screening in this age group. Participants with known cardiac disease were thus excluded [i.e. VHD, ischaemic heart disease (IHD), atrial fibrillation (AF), and those with clear symptomatic or past history of heart failure]. In this way, we selected only healthy asymptomatic subjects without an indication for echocardiography according to contemporary guidelines and practice.

For data collection, a questionnaire was used to determine the presence of cardiovascular risk factors including hypertension, diabetes, and smoking. Participants were also assessed for symptoms of dyspnoea using the New York Heart Association classification, chest pain, and palpitations. The clinical examination included measurements of height, weight, heart rate (HR), and blood pressure (BP). The transthoracic echocardiograms followed a standardized protocol that had been agreed upon before patient recruitment began and followed by all sites, which included assessments of left ventricular ejection fraction (biplane Simpson’s), left atrial area and volume, left ventricular wall thickness, diastolic parameters (*E*/*A* ratio, deceleration time, mitral inflow propagation velocity, pulmonary vein flow, and tissue Doppler velocities), and valvular function. Valvular disease severity was determined using European Association of Cardiovascular Imaging criteria.^18–21^ Any cases undertaken before the publication of these guidelines were retrospectively quantified against these guidelines. The presence of trivial valvular regurgitation and that of aortic sclerosis were considered to be part of the normal spectrum and thus not included in the VHD group. The vast majority of the scans were performed at the local general practitioner surgeries, by two experienced and accredited sonographers, while some were conducted in the hospital setting by the same sonographers if, for example, there was no suitable room for performing echocardiography in the general practice.

The study was approved by the NHS England Research Ethics Committee, received institutional approval from all three areas of the UK, and was completed according to the ethical standards of the Declaration of Helsinki. All participants provided written informed consent. The STROBE (STrengthening the Reporting of OBservational studies in Epidemiology) statement was used in the conduct and reporting of this study.^22^

### Statistical analysis

The participants were classified according to demographic characteristics, clinical examination data, and presence of cardiovascular risk factors. Descriptive statistics for the study cohort are presented using means and standard deviation (SD) for continuous variables and counts (percentages) for categorical variables. Student’s *t*-test and *χ*^2^ test or Fisher’s exact test were used to explore associations between VHD and quantitative and categorical variables, respectively. For multivariate regression, all variables with initial univariate regression *P*-value ≤0.05 were included and subsequently removed if the Wald test *P*-value was ≤0.05. All results are expressed as odds ratios (ORs) with 95% confidence intervals (CIs), and a two-tailed *P*-value ≤0.05 was considered significant. The STATA Statistical software^23^ was used for all statistical analyses performed.^24^

## Results

### Demographics

Of the 10 000 people over 60 years of age who were invited to participate in the study, a total of 5429 subjects volunteered. Of these, 881 (16.2%) were identified with known VHD, heart failure, IHD, or AF on the day of the scheduled echocardiogram and were thus excluded. The number of participants who had echocardiograms deemed to be of adequate quality for the assessment of valve function was 4237 (93.2%; see Supplementary data online, *Figure S1*). The mean age was 69.1 (±6.73) years, 54.2% were female, and the vast majority of the cohort was of White ethnic background (97.7%) in line with the UK demographics in this age group. Baseline participant characteristics are detailed in *Table 1*.

**Table 1:** Baseline study population characteristics in relationship to the presence of valvular disease

	All patients *n* (% or SD)	Valvular disease absent *n* (% or SD)	Valvular disease present *n* (% or SD)	*P*-value
Number of patients	4237 (100)	3042 (71.8)	1195 (28.2)	
Demographics				
Mean age (years)	69.1 (±6.73)	68.4 (±6.4)	70.9 (±7.3)	**<0**.**001**
Male	1941 (45.8)	1412 (46.4)	529 (44.3)	0.21
Female	2296 (54.2)	1630 (53.6)	666 (55.7)	
Ethnicity				
White	4146 (97.9)	2974 (97.8)	1172 (98.1)	0.70
Asian	78 (1.8)	59 (1.9)	19 (1.6)	
Afro-Caribbean	13 (0.3)	9 (0.3)	4 (<0.1)	
Clinical examination				
SBP (mmHg)	142.8 (±19.8)	142.1 (±19.6)	144.2 (±20.6)	**0**.**005**
DBP (mmHg)	79.5 (±10.6)	79.8 (±10.5)	77.6 (±10.7)	**0**.**002**
HR (bpm)	70.7 (±12.1)	71.6 (±12.1)	68.5 (±12.1)	**<0**.**001**
BMI (kg/m^2^)	26.9 (±4.7)	27.1 (±4.6)	26.4 (±4.4)	**<0**.**001**
Cardiovascular comorbidities and risk factors				
Hypertension	1544 (36.4)	1066 (35.0)	478 (40.0)	**0**.**003**
Hypercholesterolaemia	1032 (24.4)	716 (23.5)	316 (26.4)	**0**.**047**
Diabetes mellitus	332 (7.8)	247 (8.1)	85 (7.1)	0.27
Smoking	410 (9.7)	334 (11.0)	76 (6.4)	**<0**.**001**
Medications				
Aspirin	514 (12.1)	352 (11.6)	162 (13.6%)	0.07
Clopidogrel	57 (1.3)	35 (1.2)	22 (1.8)	0.08
Beta-blockers	383 (9.0)	240 (7.9)	143 (12.0)	**<0**.**001**
Calcium channel blockers	468 (11.0)	322 (10.6)	146 (12.2)	**0**.**12**
ARBs	218 (5.1)	137 (4.5)	81 (6.8)	**0**.**003**
ACEIs	531 (12.5)	390 (12.9)	141 (11.8)	0.37
Statins	925 (21.8)	647 (21.3)	278 (23.3)	0.15
Diuretics	537 (12.7)	381 (12.6)	156 (13.1)	0.63

Bold values indicate statistical significance. *P*-value was determined using Student’s *t*-test and *χ*^2^ test for quantitative variables and Fisher’s exact test for categorical variables.

NYHA, New York Heart Association; SBP, systolic blood pressure; DBP, diastolic blood pressure; HR, heart rate; BMI, body mass index; ARBs, angiotensin II receptor blockers; ACEIs, angiotensin-converting enzyme inhibitors.

### Patient history and clinical characteristics

Of the 4237 individuals who were included in the study, 1195 (28.2%) participants were found to have VHD (aortic sclerosis and any trivial regurgitation were excluded). The most commonly present modifiable cardiovascular risk factors were hypertension (HTN; 36.4%), hypercholesterolaemia (24.4%), smoking (9.7%), and diabetes mellitus (7.8%). The median arterial BP was 142.8/79.5 mmHg with a median HR of 70.7 bpm. The mean body mass index (BMI) was 26.9 (±4.7) (kg/m^2^), and 70.8% of participants were overweight. Subjects with valvular pathology were significantly older, were more frequently hypertensive or hypercholesterolaemic, had lower BMI, and were less frequently smokers.

Mean systolic BP was higher in the VHD group (144.2 vs. 142.1 mmHg, *P* = 0.005), but diastolic BP was lower (77.6 vs. 79.8 mmHg, *P* = 0.002). Participants with VHD also had a lower mean HR (68.5 vs. 71.6 bpm, *P* < 0.001). Interestingly, the mean BMI was also lower in the VHD group (26.4 vs. 27.1 kg/m^2^, *P* < 0.001; *Table 1*).

### Prevalence of valve disease

The prevalence rate of any newly diagnosed VHD in the study population was 28.2% (*n* = 1195). This included mild, moderate, or severe VHD but not trivial valvular regurgitation or aortic sclerosis as per our design.

**Figure 1:**
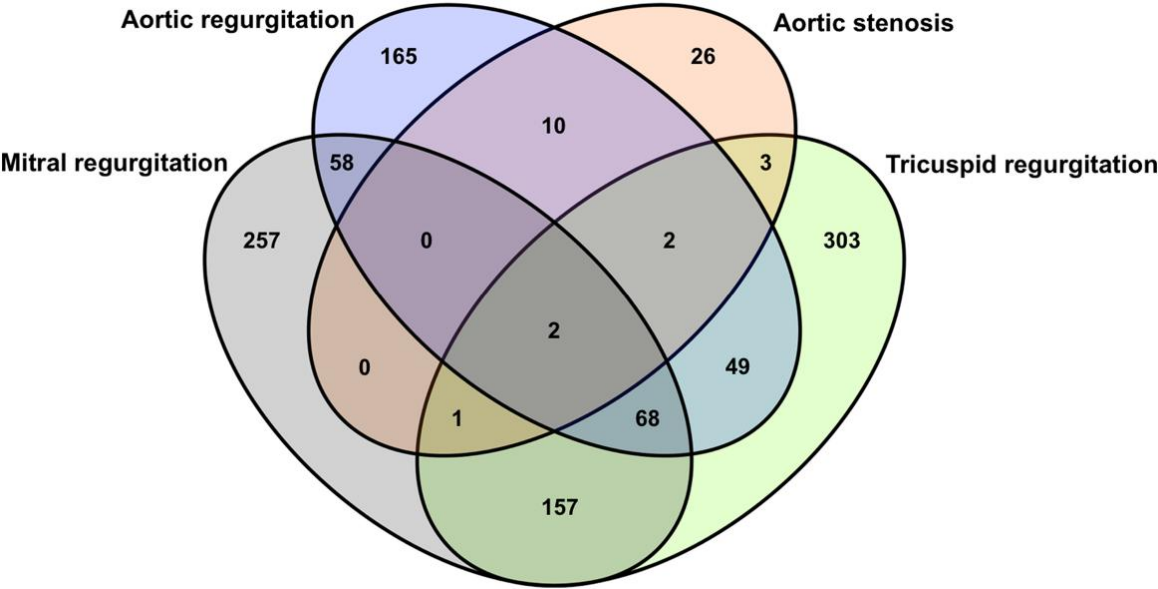
A Venn diagram demonstrating the prevalence of isolated and combined valve disease.

In order of frequency, isolated or combined tricuspid regurgitation (TR; *n* = 585, 13.8%) was the most frequent valve abnormality in the study population, followed by mitral regurgitation (MR; *n* = 543, 12.8%), aortic regurgitation (AR; *n* = 354, 8.3%), and pulmonary regurgitation (PR; *n* = 253, 6.0%). Aortic stenosis, either alone or in combination with other lesions, was the most common stenotic lesion affecting 44 (1.0%) patients of the cohort, while mitral stenosis was found in 4 (0.1%). No cases of pulmonary or tricuspid stenosis were identified (*Table 2*). The presence of isolated single-valve disease accounted for 63.6% of the cases, as shown in the Venn diagram in *Figure 1*. Incidental cardiac tumours were discovered in three participants (two atrial myxomas and one aortic fibroelastoma).

**Table 2:** Prevalence and severity of valve disease in a subgroup of patients without known cardiac disease

Type and severity of valvular disease % of total population	Mild	Moderate	Severe	Total
Any valve disease	1094 (25.8%)	94 (2.2%)	7 (0.2%)	1195 (28.2%)
Aortic stenosis	21 (0.5%)	18 (0.4%)	5 (0.1%)	44 (1.0%)
Aortic regurgitation	324 (7.6%)	30 (0.7%)	0	354 (8.3%)
Mitral stenosis	3 (0.1%)	1 (<0.1%)	0	4 (0.1%)
Mitral regurgitation	508 (12.0%)	33 (0.8%)	2 (<0.1%)	543 (12.8%)
Tricuspid regurgitation	564 (13.3%)	21 (0.5%)	0	585 (13.8%)
Pulmonic regurgitation	251 (5.9%)	2 (<0.1%)	0	253 (6.0%)

Classification determined according to the most severe lesion.

Clinically significant valve disease (moderate or severe) was present in only 101 participants (2.4%), indicating that 42 echocardiographic studies were needed to identify one patient with significant disease. The most commonly occurring moderate or severe lesions were mitral and aortic regurgitation, followed by tricuspid regurgitation and aortic stenosis (*Table 2*). Of the 101 patients with significant VHD, only 7 had severe VHD (5 AR and 2 MR).

The causes of the significant valve disease were further analysed. Of the 34 patients with moderate or severe MR, information on the cause could be determined in 23 of them. Coaptation defect secondary to annular dilatation was the main cause of MR in eight patients, annular calcification in seven, mitral valve prolapse in seven, and functional (due to heart failure) in one patient. The only case of a patient with moderate mitral stenosis was attributed to degenerative calcification.

All cases of patients with moderate or severe aortic stenosis (*n* = 23) were attributed to calcific degeneration. Among the 30 participants with moderate AR, the cause was determined in 26 patients. In 19 patients, this was due to degenerative calcification, and root dilatation was the cause in 7 patients.

### Parameters associated with VHD

From the total population of 4237 patients, 2490 patients were 60–69 years old, 1378 patients were 70–79 years old, 354 patients were 80–89 years old, and 15 patients were 90 years old or older. The number of patients in each group, along with the number of participants with moderate or severe VHD in each group, is given in *Table 3*.

**Table 3:** Total number of patients and number of patients with moderate or severe VHD in each age group

Age group	Number of patients, *n* (%)	Patients with moderate or severe VHD, *n* (%)
60–69	2490 (58.7)	44 (1.7)
70–79	1378 (32.5)	38 (2.7)
80–89	354 (8.4)	18 (5)
≥90	15 (0.3)	1 (6.7)

**Figure 2:**
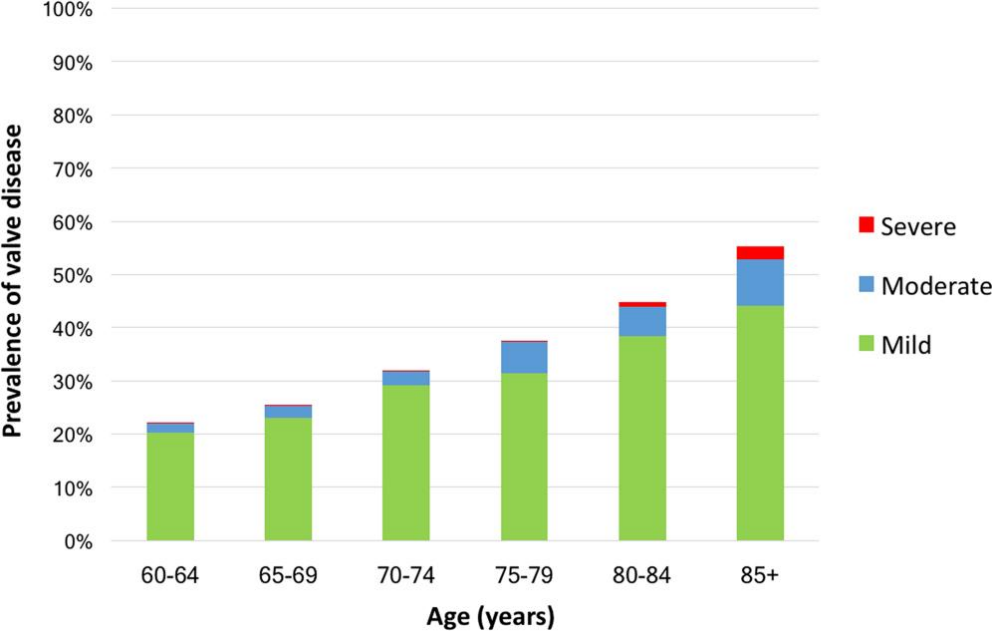
Prevalence of heart valve disease with increasing age.

Participants with VHD were on average 2.5 years older than those without VHD (*P* < 0.001). The prevalence rate of VHD increased with age, from 21.2% in those aged 60–64 to 31.5% in those aged 70–74, and 53.6% in those over 85 years old (*Figure 2*). As such, if we focus on the group of patients who are 75 years old or more, 15 scans are needed to diagnose one patient with clinically significant VHD.

**Figure 3:**
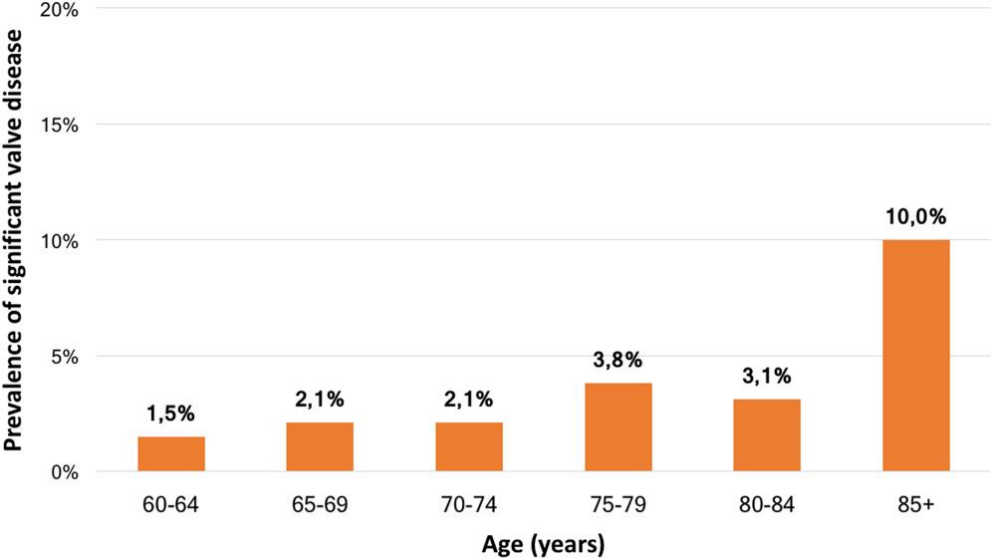
Prevalence of significant valve disease (defined as moderate or severe) with increasing age.

Multivariate regression analysis identified age as the only parameter associated with clinically significant (at least moderate) VHD (OR 1.07 per 1 year increment, 95% CI 1.05–1.09, *P* < 0.001). Indeed, the prevalence rate of significant VHD increases with age, reaching 10% in participants over 85 years old, as shown in *Figure 3*.

Pulse pressure was shown to have an association with the presence of clinically significant VHD in univariate analysis, but the significance of this correlation was lost in multivariate regression analysis (OR 1.01, 95% CI 0.99–1.02, *P* = 0.16). The presence of comorbidities, including HTN, AF, IHD, hypercholesterolaemia, and heart failure, as well as sex and BMI, were tested, but none was found to be associated with the presence of moderate or severe VHD (multivariable regression is shown in Supplementary data online, *Figure S2*).

## Discussion

This study represents the largest prospective community-based echocardiographic study assessing the prevalence of VHD in asymptomatic subjects of 60 years old or more with no pre-existing cardiac conditions. More than a quarter of the total population (28.2%) were diagnosed with VHD, the most common being mild tricuspid, mitral, and aortic regurgitation. Age was found to be significantly associated with the presence of VHD, with a higher incidence of at least moderate VHD in individuals of 75 years old or more.

The results of this study reflect that asymptomatic VHD is present in a significant proportion of people aged 60 years old or more. This finding is in agreement with previously published data, although arguably, the prevalence reported in this study is lower than what has previously been reported.^7,25^ In one of the largest to-date community-based studies, the OxVALVE cohort study, D’Arcy *et al*.^25^ found that the prevalence rate of asymptomatic and previously undiagnosed VHD was just over 50% in a population that included 2500 participants. While both our study and the OxVALVE study highlight the high burden of VHD in the community, the differences in the percentage of patients with VHD potentially reflect the differences in the inclusion criteria and the baseline characteristics of the participants. In the present study, individuals with aortic sclerosis were not included, while in the OxVALVE study, all subjects with aortic sclerosis were included, and this pathology was the most commonly encountered (34% of the patients with newly diagnosed VHD).^25^ In addition, the OxVALVE study identified eight subjects (0.3%) with bicuspid aortic valve disease, but patients with known valve disease had also been included. In our cohort, we did not identify anyone with bicuspid aortic valve disease, presumably because they would have had symptoms or identified clinically before the age of 65 and thus become ineligible for our study. Furthermore, subjects with AF were excluded from this study. This is in contrast to the OxVALVE study, in which AF was found to be the variable most strongly correlated with newly diagnosed clinically significant VHD.^25^ In addition, the mean age of the participants of the present study was lower than that of the participants of the OxVALVE study (69.1 vs. 74.2 years old, respectively). This is an important consideration, as the prevalence of VHD increases with age.^7,26,27^

Our study demonstrated that the presence of VHD is strongly associated with age, with the incidence of VHD being more than double in those over 85 years old compared with those <70 years of age. This finding is consistent with previous data, and also with the pathophysiological theory that identifies increased haemodynamic stress over the years as an important factor of valve degeneration with age.^4,7,8,25–27^ Importantly, the prevalence rate of clinically significant (at least moderate) VHD in this study was 2.4%, with age being the only important parameter associated with its presence. Indeed, for subjects over 75 years, the prevalence rate of clinically significant VHD increased to 6.7%. Not only is clinically significant VHD of paramount importance for early risk stratification of patients, but it has also been shown to be associated with increased mortality and morbidity in the elderly population.^28^ In a population-based study that included more than 500 individuals ≥80 years old, Rezzoug *et al*.^28^ found an increased rate of prevalence (17%) of moderate-to-severe VHD in this cohort, with age being an independent predictor of all-cause mortality.

Based on the results of our study, 15 echocardiographic studies are needed to diagnose one individual with clinically significant VHD in patients ≥75 years old. It is important to note, however, that only 21% of the total population (900 of the 4237 patients) of this study were 75 years old or older and only 8.6% were ≥80 years old. In this era of cost-effectiveness, and focused echocardiography using portable echocardiographic machines, these figures can be considered by policymakers to identify whether screening for heart valve disease can be made more cost-effective.

The analysis of this uniquely selected large study cohort demonstrates that VHD is present in a significant proportion of asymptomatic and otherwise considered healthy individuals over 60 years old, and its prevalence increases with age. Clinically significant VHD is more common across the elderly population, and larger epidemiological studies are needed to clarify the disease burden in this specific cohort and the role of screening echocardiography in risk stratification and management strategies.

### Limitations

One limitation of the study stems from the fact that participation was voluntary. Although more than 50% of individuals chose to participate, the risk of selection bias persists, with the more ‘unwell’ patients not taking part, a problem inherent with community studies.^25,29,30^ In addition, we recruited patients only from three counties of the UK, which again can lead to bias, although to mitigate this, the general practice surgeries included in this research were chosen to be representative of the general population in terms of ethnicity and affluence.

Moreover, we do not have b-type natriuretic peptide (BNP)/N-terminal proBNP values in our patients, as this study was initiated prior to natriuretic peptides becoming clinically relevant and widely available. Therefore, we are unable to comment on whether an initial BNP test could have identified patients who might have benefited from an echocardiogram. Furthermore, the vast majority of the cohort was of white ethnic background (97.7%), which is, however, consistent with the most recent census data that show that people over 60 years old are 95% of white ethnicity in England and 99% in Scotland.^31,32^ Still, due to this large ethnic homogeneity, it is unclear whether these findings can be generalized to other communities. In addition, while we do not have information of patients with potentially ‘innocent’ murmurs on the day of echocardiography, it has been demonstrated that cardiac auscultation has limited accuracy in primary care.^9,33^

Finally, while our study identifies the numbers needed to scan, it has not been designed to evaluate the implementation of such a programme, which may be even more challenging with an increasingly ageing population.

## Conclusion

This study represents the largest prospective community-based study, assessing the prevalence of asymptomatic VHD in individuals of 60 years of age or older. We demonstrate that VHD is present in a significant proportion of otherwise considered healthy individuals over 60 years old. Age is strongly associated with the incidence of VHD, including clinically significant VHD, which is more prevalent in the elderly population. These valuable data can lay the foundation for further epidemiological studies evaluating the burden of VHD in the community and the potential role of echocardiographic screening in the elderly population.

## Ethics approval

The study was approved by the NHS England Research Ethics Committee, received institutional approval from all three areas of the UK, and was completed according to the ethical standards of the Declaration of Helsinki.

## Consent to participate

All patients provided written informed consent.

## Consent for publication

All patients provided written informed consent.

## Supplementary Material

jeae127_Supplementary_Data

## Data Availability

All data are available from the corresponding author on request.
