# Supplementary material for: Prevalence of asymptomatic valvular heart disease in the elderly population: a community-based echocardiographic study
Source: Eur Heart J Cardiovasc Imaging. 2024 Jun 26;25(8):1051–8. doi: 10.1093/ehjci/jeae127 (PMC11288748; doi:10.1093/ehjci/jeae127)
Supplement: jeae127_Supplementary_Data [file jeae127_supplementary_data.docx]

**Supplementary Material**

**Prevalence of asymptomatic valvular heart disease in the elderly population: a community-based echocardiographic study**

Vasiliki Tsampasian MSc MRCP^1,2^, Cristian Militaru MD PhD^1^, Sathish Kumar Parasuraman MBBS MRCP^1^, Brodie L Loudon MBBS^1^, Crystal Lowery BSc MA^1^, Amelia Rudd BSc^3^, Janaki Srinivasan BSc^4^, Satnam Singh MBBS MRCP^5^, Girish Dwivedi DM MRCP PhD^6^, Gnanadevan Mahadavan MBBS PhD^7^, Dana Dawson DM FRCP D.Phil FESC^3^, Allan Clark BSc PhD^1^, Vassilios S. Vassiliou MBBS PhD FESC FACC^1,2#^, Michael P. Frenneaux MD FRCP FRACP FACC FESC^8#^

**eTable. Prevalence and severity of valve disease in subgroup of patients without known cardiac disease.**

**eFigure1. Flow diagram for participant selection**

**eFigure2. Multivariable regression analysis of possible factors associated with the presence of VHD.**

**eTable. Prevalence and severity of valve disease in subgroup of patients without known cardiac disease.** Classification determined according to the most severe lesion.

| **Type and severity of valvular disease**  **% = of total population** | **Mild** | **Moderate** | **Severe** | **Total** |
| --- | --- | --- | --- | --- |
| Any valve disease | 1094 (25.8%) | 94 (2.2%) | 7 (0.2%) | 1,195 (28.2%) |
| Aortic stenosis | 21 (0.5%) | 18 (0.4%) | 5 (0.1%) | 44 (1.0%) |
| Aortic regurgitation | 324 (7.6%) | 30 (0.7%) | 0 | 354 (8.3%) |
| Mitral stenosis | 3 (0.1%) | 1 (<0.1%) | 0 | 4 (0.1%) |
| Mitral regurgitation | 508 (12.0%) | 33 (0.8%) | 2 (<0.1%) | 543 (12.8%) |
| Tricuspid regurgitation | 564 (13.3%) | 21 (0.5%) | 0 | 585 (13.8%) |
| Pulmonic regurgitation | 251 (5.9%) | 2 (<0.1%) | 0 | 253 (6.0%) |

**eFigure1. Flow diagram for participant selection**


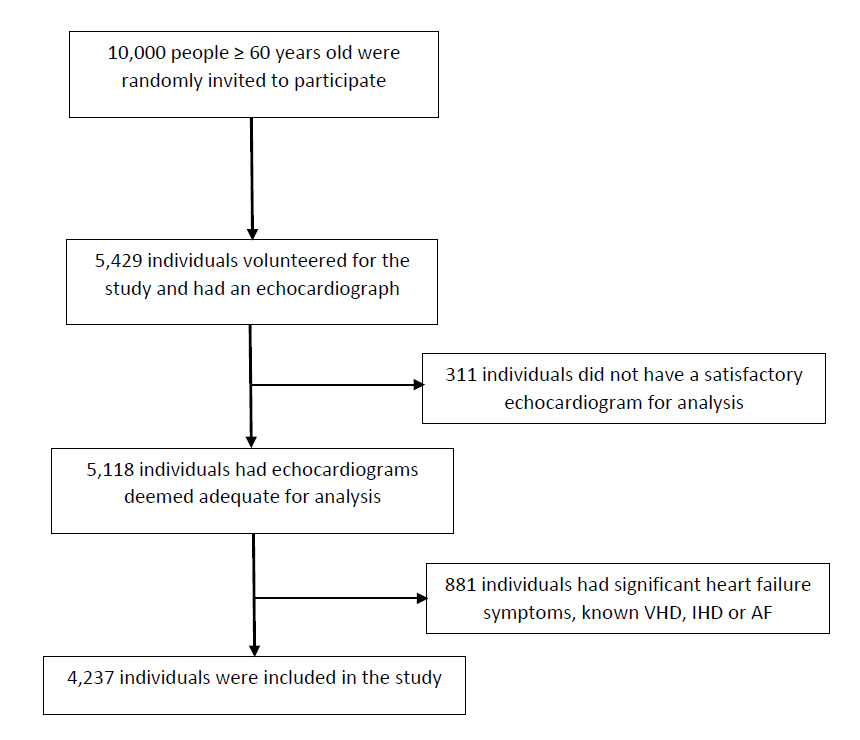


**eFigure2. Multivariable regression analysis of possible factors associated with the presence of VHD.** Only age was found to be significantly associated with VHD.
